# Identifying Factors Which Influence Eating Disorder Risk during Behavioral Weight Management: A Consensus Study

**DOI:** 10.3390/nu15051085

**Published:** 2023-02-22

**Authors:** Hiba Jebeile, Caitlin M. McMaster, Brittany J. Johnson, Sarah P. Garnett, Susan J. Paxton, Anna L. Seidler, Rebecca A. Jones, Andrew J. Hill, Sarah Maguire, Caroline Braet, Genevieve Dammery, Denise E. Wilfley, Louise A. Baur, Natalie B. Lister, on behalf of the Eating Disorders In weight-related Therapy (EDIT) Collaboration

**Affiliations:** 1 Children’s Hospital Westmead Clinical School, The University of Sydney, Sydney 2145, Australia; 2 Charles Perkins Centre, The University of Sydney, Sydney 2006, Australia; 3 Caring Futures Institute, College of Nursing and Health Sciences, Flinders University, Adelaide 5042, Australia; 4 Institute of Endocrinology and Diabetes, The Children’s Hospital at Westmead, Sydney 2145, Australia; 5 Kids Research, The Children’s Hospital at Westmead, Sydney 2145, Australia; 6 School of Psychology and Public Health, La Trobe University, Melbourne 3086, Australia; 7 National Health and Medical Research Council Clinical Trials Centre, The University of Sydney, Sydney 2050, Australia; 8 MRC Epidemiology Unit, University of Cambridge, Cambridge CB2 0QQ, UK; 9 Leeds Institute of Health Sciences, University of Leeds, Leeds LS2 9JT, UK; 10 InsideOut Institute for Eating Disorders, Charles Perkins Centre, The University of Sydney, Sydney 2006, Australia; 11 Department of Developmental, Personality and Social Psychology, Ghent University, 9000 Ghent, Belgium; 12 School of Medicine, Washington University in St. Louis, St Louis, MO 63110, USA; 13 Weight Management Services, The Children’s Hospital at Westmead, Sydney 2145, Australia

**Keywords:** disordered eating, dieting, intervention strategies, delivery features, behavior change, obesity, overweight

## Abstract

This study aimed to understand clinician, researcher and consumer views regarding factors which influence eating disorder (ED) risk during behavioral weight management, including individual risk factors, intervention strategies and delivery features. Eighty-seven participants were recruited internationally through professional and consumer organizations and social media and completed an online survey. Individual characteristics, intervention strategies (5-point scale) and delivery features (important/unimportant/unsure) were rated. Participants were mostly women (n = 81), aged 35–49 y, from Australia or United States, were clinicians and/or reported lived experience of overweight/obesity and/or ED. There was agreement (64% to 99%) that individual characteristics were relevant to ED risk, with history of ED, weight-based teasing/stigma and weight bias internalization having the highest agreement. Intervention strategies most frequently rated as likely to increase ED risk included those with a focus on weight, prescription (structured diets, exercise plans) and monitoring strategies, e.g., calorie counting. Strategies most frequently rated as likely to decrease ED risk included having a health focus, flexibility and inclusion of psychosocial support. Delivery features considered most important were who delivered the intervention (profession, qualifications) and support (frequency, duration). Findings will inform future research to quantitatively assess which of these factors predict eating disorder risk, to inform screening and monitoring protocols.

## 1. Introduction

The need to consider eating disorder risk as part of weight management is increasingly being recognized [1,2,3,4]. In community samples, the development of eating disorders is influenced by multiple biological, psychological, developmental and sociocultural factors together with disordered eating behaviors [5,6,7,8]. A systematic review of 25 studies found body dissatisfaction to be the most consistent predictor of eating disorder risk in adolescents, followed by depression and low self-esteem [9]. Female sex and high body mass index (BMI) were also identified as important factors, both of which show strong associations with body dissatisfaction [9]. Adverse childhood experiences and interpersonal functioning are associated with eating disorder risk, with childhood sexual abuse and appearance-related teasing and victimization identified as having the most convincing evidence for being risk factors in an umbrella review of meta-analyses [10]. Similarly, experience of weight stigma in people with overweight or obesity is associated with disordered eating [11]. However, current literature on eating disorder risk has several limitations. Many studies are of a cross-sectional design, include females alone [9] and have predominantly been conducted in populations with an adult (or equivalent) BMI < 25 kg/m^2^. Importantly, it is unclear which factors increase risk of eating disorders in the context of weight management. Extrapolation or generalizing evidence of eating disorder development to the context of weight management should be approached with caution. Some known eating disorder risk factors may be less applicable, or additional risk factors specific to weight management may not yet be identified. If there are additional factors specific to weight management not captured by current literature, these should be identified so that preventative measures can be put in place.

Behavioral weight management interventions are often first-line treatment for overweight or obesity [12,13,14,15]. Although interventions tend to include a combination of dietary modification, physical activity, sleep and behavior change strategies, a large degree of heterogeneity exists between interventions. Evidence from systematic reviews demonstrate that behavioral weight management can support weight loss for up to two and five years, in adolescents and adults, respectively [16,17]. However, some common components of weight management may be risk factors for eating disorders. For example, caloric restriction and reduced intake of energy-dense foods are often recommended as part of weight management interventions [18], although dietary restraint is an established risk factor for binge eating [5]. Similarly, self-monitoring of weight or caloric intake, while beneficial for weight loss outcomes in weight management settings [19], is associated with increased disordered eating in community samples [20]. Other strategies, such as promoting regular meal-time routines, goal setting and family-based treatment are used both in weight management and to address disordered eating [21]. Thus, to improve our understanding of the intersection between behavioral weight management and eating disorder risk, it is important to be able to differentiate between intervention strategies likely to modify eating disorder risk in this context.

The Eating Disorders In weight-related Therapy (EDIT) Collaboration aims to explore the complex risk factor interactions that may precede changes in eating disorder risk during behavioral weight management interventions (www.editcollaboration.com; accessed on 17 November 2022) [22]. Specifically, the EDIT Collaboration seeks to identify early individual predictors of eating disorder risk and understand which components of weight management interventions may contribute to change in eating disorder risk. EDIT is the first program of research to examine eating disorder risk at the individual level during weight management interventions. There may be individual risk factors or intervention strategies relevant to this context not previously examined in the literature. Indeed, expert and lived experience opinion can provide important insights in setting research agendas to address such research gaps. To capture these potentially missing contributors, we aimed to understand clinician, researcher and consumer views regarding which individual characteristics may increase risk of eating disorders in the context of weight management, and which intervention strategies may increase or decrease risk of eating disorders. The study aim was to synthesize the views of these groups and better understand how individual characteristics and intervention strategies may influence eating disorder risk during weight management interventions.

## 2. Materials and Methods

### 2.1. Study Design and Participants

This study had a cross-sectional design, with an online survey administered on Qualtrics software (Qualtrics, Provo, UT, USA). Target participants were adults aged ≥18 years with clinical, research and/or lived experience of an eating disorder and/or overweight and obesity. The survey was first sent by email to members of the EDIT Collaboration, which includes an international membership of clinicians, researchers and stakeholders working across the fields of obesity and eating disorders. Additional participants were recruited internationally via advertisement through professional societies and advocacy associations representing consumers with lived experience, including Australia New Zealand Obesity Society, Australia New Zealand Academy for Eating Disorders, Dietitians Australia, National Eating Disorder Collaboration, Academy for Eating Disorders, European Association for the Study of Obesity, British Dietetic Association, Obesity Action Coalition, InsideOut Institute for Eating Disorders, Weight Issues Network and The Obesity Collective. Organizations advertised the survey to members either via a newsletter, through a discussion forum, website listing, interest group, or on social media. The survey was also advertised on social media by study investigators. Snowball sampling was used by asking participants to distribute the survey with colleagues. Data collection occurred between 7 February and 6 March 2022. The study was approved by the Human Research Ethics Committee of The University of Sydney [2021/822]. We aimed for a sample size of 60 to 100 participants, with at least 20 participants from each target population (clinicians, researchers and lived experience), to allow for a broad range of views to be captured across the target populations. Participants provided informed consent online when initiating the survey and by returning a partial or completed survey response. All responses were anonymous. Fraud detection functions available as part of Qualtrics software were used to detect possible duplicate responses, and those from bots (multiple responses, often from a software program) were detected using reCAPTCHA technology.

### 2.2. Survey Development and Data Collection

A list of individual characteristics potentially relevant to eating disorder risk and common components of weight management interventions were drafted based on the literature and the study team’s experience with weight management trials. Nine clusters of individual characteristics were drafted, including participant demographics and weight status, general medical history, weight-related medical history, eating-disorder-related medical history, mental-health-related medical history, psychosocial health, eating behaviors and history of dieting.

Intervention components were categorized as delivery features and intervention strategies. Delivery features were defined as “a broad number of intervention characteristics that relate to how an intervention is delivered” [23] and were adapted from the Template for Intervention Description and Replication checklist [24]. Delivery features included the target population, who delivered the intervention, mode of delivery, intervention setting and the number and range of outcome assessments used within an intervention. Intervention strategies describe the behavior change content of weight management interventions. Intervention strategies were first grouped into five broad categories (intervention intent, framing and outcomes; dietary strategies; eating behaviors and disordered-eating-related strategies; movement and sleep-related strategies; and psychosocial-health-related strategies) and then as clusters of unique, related strategies within each category. Each category included several clusters.

The individual characteristics and intervention components were refined through an iterative consultation process with the EDIT Collaboration Scientific and Stakeholder Advisory Panels via four online workshops (June 2021). At each workshop, the items within each category were discussed, new items were added, and similar items were combined or grouped. Feedback from each workshop was included in subsequent workshops. Finally, the list of individual characteristics and intervention components were further refined to remove repetition, ensure consistent language and address clustering.

The survey was available in English and was estimated to take 30 to 40 min to complete. Participants’ demographics (including age, gender and ethnicity) and clinical, research and lived experience with eating disorders and/or overweight or obesity were captured. The survey had three parts:

1. Individual characteristics—Participants were asked to rate the relevance of individual participant characteristics to the risk of developing an eating disorder in the context of weight management interventions. Items were rated on a five-point Likert scale from 1 = not relevant at all to 5 = very relevant. Participants were prompted to add individual characteristics not already included as free text.
2. Intervention strategies—Participants were asked to rate various strategies used during weight management interventions as to whether they would likely increase, decrease or have no impact on eating disorder risk. Items were rated on a five-point Likert scale from 1 = very likely to reduce eating disorder risk to 5 = very likely to increase eating disorder risk. Participants were prompted to add intervention strategies not already included as free text.
3. Delivery features—Participants were asked to rate the importance (important, not important, unsure) of key delivery features in relation to eating disorder risk during weight management interventions. Participants were prompted to add delivery features not already included as free text.

At the end of the survey, participants were given the opportunity to provide additional comments on eating disorder risk during weight management interventions.

#### Content Validity and Pilot Testing

Content validity was assessed by expert review of the survey instrument by a clinician, researcher and an individual with lived experience of eating disorders from within the EDIT Collaboration. Reviewers were asked to comment on the survey’s content and wording of questions and scales in relation to the aims of the survey. The survey was subsequently updated based on this feedback. The online version of the survey was then pilot tested by five stakeholders not involved in survey development (from within and external to the EDIT Collaboration), including four clinicians and/or researchers working in obesity, eating disorders, or both, and a person with lived experience of obesity. The pilot sample was asked to identify any errors or barriers in form and presentation of the survey and use of the online system.

### 2.3. Analysis

Descriptive statistics were used to summarize demographic data and frequency of responses for Likert scale questions using SPSS version 27 (IBM SPSS Statistics for Windows, Version 27.0. Armonk, NY, USA: IBM Corp). We considered possible variations in response based on professional group (clinician, researcher) and/or lived experience; however, this was not possible, as most respondents reported experience across groups. Free-text responses suggesting additional items to be considered and general comments were collated in Microsoft Excel. Responses were independently coded by two authors and grouped into broad themes, with consensus achieved through discussion (HJ and CMM).

## 3. Results

There were 121 participants who provided consent and initiated the survey. Of these, one record was identified as a duplicate by Qualtrics and removed, and 33 records were excluded, as no questions other than in the demographics section were completed. The remaining 87 responses were included in analyses. There were no statistically significant differences in age, gender, professional background or discipline of those who completed the survey and those who completed the demographic section only.

### 3.1. Participant Demographics

Characteristics of respondents are summarized in Table 1. Most respondents were women (n = 81, 93%) aged 35 to 49 years (n = 35, 40%). Most (n = 71, 82%) reported lived experience of high weight, eating disorders and/or were carers of a person with higher weight or an eating disorder (n = 22, 25% higher weight alone; n = 12, 14%, any eating disorder alone). Twenty-five participants (29%) reported lived experience of both higher weight and eating disorders (Table 1).

### 3.2. Individual Characteristics

**Figure 1:**
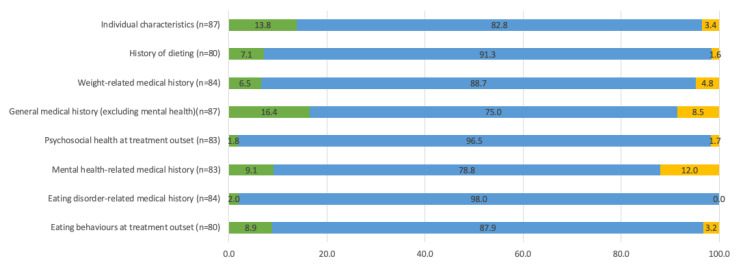
Summary of clusters of individual characteristics (n = 87). Items were rated on a five-point Likert scale from 1 = not relevant at all to 5 = very relevant. See Table S1 for ratings of individual items within each cluster.

Across all nine clusters of individual characteristics, there was agreement (64% to 99%) that these were somewhat likely, likely or very likely to be relevant to eating disorder risk in the context of weight management (Figure 1, Table S1). Age at menopause (unlikely 35%, likely 47%, not sure 18%) and diagnosis of oppositional defiant disorder (23%, 44.5%, 32.5%) had a greater proportion of responses as unlikely to be relevant to eating disorder risk or not sure. Additional individual characteristics which may be relevant to eating disorder risk in the context of weight management, identified by stakeholders, are summarized in Table 2. Additional items suggested included consideration of genetic risk factors, mental health comorbidity (e.g., history of addiction, self-harm, poor executive function), diagnosis of obstructive sleep apnea, emotional response to dieting attempts and weight loss, use of medications that increase or decrease appetite regulation, social media use and the influence of the family and environmental context on the individual. It was also suggested that risk factors may vary by type of eating disorder and that disorder-specific risk should be considered.

A common view among participants was that “weight-related attitudes of family and professionals involved in care” and experience of weight stigma and “fat phobia” from health professionals are important to consider. These include, for example, “exposure to self-disparaging comments about weight, especially from parents,” “levels and experiences of weight stigmatizing micro aggressions resulting in shame/guilt” and “how often has medical care been withheld until the individual loses enough weight?” This may appear as “micro aggressions” from staff, e.g., “You want to be healthy, right.” It was also suggested that the “power imbalance between the health professional giving the weight management advice” [and the patient] is particularly important “where clients have an identity that relates to historically significant minority groups.”

Participants suggested that eating disorder risk factors should be considered within an individual’s personal context, for example, “all of the above need to say ‘it depends’ none of these can be answered for groups, only individuals,” and “all of these need to be considered in relation to the personal context in which a person lives e.g., they may have these risks but live in a protective family, community or wider society or they may be in an unsafe environment e.g., thin ideal expectations.” This was also referred to as the “psychological resilience of the family.”

### 3.3. Intervention Strategies Used during Weight Management Interventions

#### 3.3.1. Intervention Framing and Outcomes

Intervention strategies relating to the framing of the weight management intervention (Table S2) were rated as being more likely to increase eating disorder risk if they focused on weight-related outcomes, e.g., ‘aims for weight loss’ (83% rated as more likely to increase risk) or ‘use of weight-focused language during the intervention’ (75%). Strategies relating to broader aspects of health were rated as being more likely to decrease eating disorder risk, e.g., ‘measures mental health outcomes’ (62% rated as more likely to decrease risk) and provides ‘education that health outcomes are not dependent on weight’ (73%). It was suggested that the rate of weight loss is an important consideration, with faster weight loss suggested to increase risk.

Communication and education approaches used during interventions were suggested as being important to consider in relation to eating disorder risk (Table 2). For example, it was suggested that the “name of the program and language/images used in program material, comparison to others, expectations, ‘success’ stories” and having a “decreased emphasis on personal responsibility—somewhat likely to decrease risk.” Similarly, “explaining the science of weight/appetite regulation and weight stigma” was suggested to decrease eating disorder risks, as does focusing on self-esteem, body acceptance and acting to “frame weight loss as a path to health and longevity.” It was suggested to “frame weight change as a potential/possible side effect of behavior change, rather than as a focus of behavior change. Focus on improved ability to do or engage in the things that are important to their quality of life” and to provide patient choice in the selection of a weight management approach, i.e., “ask the person, support the person to find their truth.” One respondent suggested that informed consent should be a routine component of weight management, asking, “do you include informed consent? With information about the low likelihood of success at maintaining long term weight loss and the metabolic harms of weight-cycling?”

In contrast, several participants suggested that the “the concept of weight management itself is problematic” and “any focus on weight or other numbers poses a significant risk” to “developing disordered eating behaviors and body image concerns.” Participants suggested that “weight management is not less harmful when delivered by medical professionals than diets that are self-directed” and that interventions should either “focus on weight or focus on health, you can’t do both at the same time.” It was suggested that clinicians provide “constant encouragement” without measurement of weight. As an alternative to weight management, “comprehensive knowledge and understanding of the Health at Every Size (HAES)^®^ paradigm” was suggested to reduce eating disorder risk.

#### 3.3.2. Dietary Strategies

Dietary prescription, categorization and monitoring were rated as being more likely to increase eating disorder risk, e.g., ‘very low energy diet’ (86% rated as more likely to increase risk) and ‘dietary monitoring—energy based’ (91%). Strategies focused on flexibility and using individualized or family-based approaches were rated as being more likely to decrease eating disorder risk, e.g., ‘flexible meal plan’ (61% rated as more likely to decrease risk) and ‘family-oriented approach to dietary change’ (64%). Use of behavior change techniques such as problem solving, goal setting and shopping support (practical support) had mixed responses, with some rating these as likely to increase risk, decrease risk or have no impact on eating disorder risk (Table S3).

Communication approaches used when talking about food were suggested to be relevant to eating disorder risk, including using dichotomizing language, e.g., “framing of foods as healthy/unhealthy” as well as the role of family dynamics, e.g., “have to be really careful about involving family/partner. If they’re overly controlling, critical or micromanaging the food purchase/intake then that would be highly likely to lead to guilt, shame, food secrecy, etc. Whole family needs to be educated about ED risk and flexible eating.” This is particularly the case if “food and selection choices different to rest of household e.g., more restrictive.”

Dietary prescription and restriction, including weighing and measuring foods, were suggested to increase eating disorder risk: “all counting, categorizing, and limiting leads straight back to disordered eating,” but “considering how different foods feel in the body, what foods provide enhanced energy levels, sit well in the stomach, keep you satiated for a long time…that’s great” (Table 2). Additionally, “household food security needs to be considered to reduce risk.”

#### 3.3.3. Eating Behaviors and Disordered-Eating Related Strategies

All eight strategies related to addressing disordered eating and promoting healthy eating behaviors, e.g., mealtime routines and mindful eating, were rated as being more likely to decrease eating disorder risk (71% to 86% rated as likely to decrease risk; Table S4). Participants suggested education on the link between eating behaviors and energy restriction and consideration of cultural context of eating behaviors as additional important strategies (Table 2).

Participants suggested screening for disordered eating during weight management; with appropriate referral and support, “will it (screening) increase the number of eating disorders you pick up in a service—yes. Will it exacerbate eating disorder symptoms in a client—no. It might help them receive appropriate and supportive treatment, but it’s unlikely to make the problem worse. Ignoring will make the eating disorder more likely.” Additionally, it was suggested that people seeking weight management may be “highly desperate for thinness and to escape stigma which makes their tolerance for risky and unsafe measures exceptionally high. They are likely to abide by the rules and feel exceptional defeat and failure when they fail to lose or begin to regain,” and that “anyone with a history of ED or currently has an ED should not be offered dieting aka weight management.” It was suggested that without appropriate identification, disordered eating may be exacerbated, e.g., “I entered the weight management program with an active eating disorder and was taught how to refine my eating disorder behaviors.” Several participants suggested that clinicians need greater awareness of and training on eating disorders and resources to be able address this as part of weight management.

#### 3.3.4. Movement and Sleep Related Strategies

Strategies based on prescriptive exercise plans or programs and self-monitoring of physical activity were rated as being more likely to increase eating disorder risk, e.g., ‘encourages strict/formal activity plan’ (80% rated as more likely to increase risk). Strategies promoting flexibility, enjoyable movement, cultural adaptations to exercise and focusing on improving sleep hygiene were rated as being more likely to decrease eating disorder risk, e.g., ‘promotes joyful movement and activity’ (82% rated as more likely to decrease risk) and ‘provides flexible exercise plan’ (69%). Strategies including use of group exercise class, personal training, education on increasing physical activity, addressing sedentary behaviors and behavior change strategies had mixed responses (Table S5). Participants suggested that addressing sleep quality (circadian alignment and total sleep time), the attitudes of personal trainers, motivations for exercise and individual traits were important considerations (Table 2), e.g., “risk associated with the personal trainer would highly depend on the attitudes and methods of the trainer….focused on developing healthy habits and increasing goals and life satisfaction, this is unlikely to increase risk…..focused on appearance management and weight loss, this would likely increase risk…..whether pedometers/monitoring tools increase risk would be very dependent on the individual and how inflexible/addictive their traits are.” It was also suggested that “encouraging people to expand their definition of what movement can entail...mental and physical health motivations for exercise vs. shape/weight/appearance” would be beneficial.

#### 3.3.5. Psychosocial Health Related Strategies

Interventions based on any of the identified psychological frameworks (55% to 75% rated as more likely to decrease risk), and those that address and monitor mental (64% to 78%) and psychosocial health (66% to 76%), weight stigma (67% to 73%) or body image (76% to 78%), and the use of behavior change strategies (67% to 80%) were rated as being more likely to decrease eating disorder risk (Table S6). Participants suggested providing education on “internalized and externally received weight biases,” “that undereating is related to anxiety, depression and difficulty concentrating,” the difficulty with long-term weight loss maintenance as the “sense of failure leads to debilitating shame” and on Health At Every Size (HAES)^®^ principles. In contrast, others suggested that it would not be appropriate to address weight stigma alongside weight management (Table 2), e.g., “Increasing the ability to recognize and managing stress/trauma related to weight stigma will only help if the person is not also consistently being told and encouraged to lose weight in a weight management setting.”

Some participants commented that although a range of individual strategies within this category were rated as being more likely to decrease eating disorder risk as part of this survey, e.g., use of cognitive behavioral therapy or family-based treatment, these would not outweigh the potential increase in risk assigned to other strategies, e.g., “I truly hope that the responses here are understood to be a non-endorsement of any weight management strategies despite certain endorsements of features discussed (e.g., intuitive eating, body appreciation, self-compassion, joyful movement, etc.).”

### 3.4. Delivery Features

Overall, all delivery features were rated as being important to consider in relation to eating disorder risk, with support provided during the intervention, e.g., frequency and duration of contact (82% rated important) and the training and qualifications (81%) of the person delivering the intervention being most important (Table S7). Participants suggested providing “step up/step down and clear pathways; continuity of care; multidisciplinary care.” The use of telehealth was raised as a potential risk factor as “there is data to show that self-focused attention during video conference calls can increase appearance concerns and drive body dissatisfaction. Telehealth delivery via videoconferencing may not be helpful for this population.”

Ensuring a safe and supportive environment “without intersectional biases” with “shared understanding of group rules and behaviors,” having “chairs that correctly and safely support (a person)” and “messaging in physical settings (i.e., signage about the ‘obesity epidemic’)” were suggested as being important. Similarly, having appropriately trained health professionals was also suggested to be important: “I do believe in body autonomy so if a client chooses to lose weight, then practitioners need to have a thorough understanding of eating disorders and body image issues,” and “for each item related to feedback on a behavior, I wasn’t sure what to choose because risk would depend on type/content of feedback.”

Participants suggested that there is high heterogeneity with how weight management intervention strategies are delivered and received by individuals. For most interventions, eating disorder risk “depends on the person,” i.e., some people will have a positive experience and others will have a stigmatizing and harmful experience with the same intervention or the same provider, e.g., “I have seen acceptance therapies promote disordered behavior, I have seen body positivity increase peoples negative self-evaluation, and I have seen them work for others. But these things that are used so often are not the answer for everyone.”

## 4. Discussion

This study aimed to understand clinician, researcher and lived experience views on the individual characteristics and intervention strategies that may contribute to eating disorder risk in the context of behavioral weight management interventions. There was broad agreement that individual risk factors, based on the existing literature, were relevant to eating disorder risk in this context. Similarly, most intervention strategies were able to be categorized as being more likely to increase or decrease risk, with few having mixed findings. There was less consensus on the perceived direction of effect for specific behavior change strategies, such as providing feedback on behavior change. Importantly, aspects of eating disorder risk unique to people with overweight or obesity and in the context of weight management were identified, including having a genetic predisposition to obesity, experiencing stigma from health professionals and having a history of bariatric surgery. Similarly, communication approaches, attitudes and beliefs of health professionals and the environmental context were identified as important components of weight management interventions to consider. This consultation process has identified new insights into the intersection between eating disorder risk and weight management interventions and will contribute to improved accuracy of assessment of eating disorder risk during clinical trials and clinical practice and to the future design of interventions.

As part of this consultation process, more than 50 individual characteristics were identified as being relevant to eating disorder risk during weight management. Many of these are consistent with the current literature on risk factors for eating disorders in the community [5,9,10]; however, additional factors specific to people with higher weight and/or the context of weight management were identified. This highlights the importance of consumer consultation when considering safety of interventions. It is important for us to understand the prevalence of these factors and the likelihood that they quantitatively predict eating disorder risk. In practice, assessing such a broad range of risk factors is resource-intensive and relies on having access to a multidisciplinary team. Additionally, many of these are not included in existing eating disorder assessments, [25] e.g., stigma from health professionals, history of bariatric surgery, weight-related teasing, childhood trauma. Thus, to facilitate routine screening and monitoring in research and clinical practice, we also need to understand which factors are most predictive of eating disorder risk in individuals undergoing weight management interventions [26]. The EDIT Collaboration is combining individual participant data from clinical trials of weight management interventions to address these research questions [27].

Our consultation process resulted in the identification of more than 100 individual components of weight management interventions (delivery features and intervention strategies). This is important because traditional evidence synthesis broadly categorizes complex interventions into sub-groups based on overarching features of interventions. For example, in a 2021 systematic review examining the effect of components of behavioral weight management on change in weight for adults, nine characteristics of interventions were considered [16]. Similarly, our 2019 systematic review examined changes in eating disorder risk during pediatric weight management and categorized the dietary strategies used in the interventions into two groups (nutrition education only or having a prescribed energy target) [28]. Yet, the present study identified 29 specific dietary strategies related to eating disorder risk. These intervention strategies varied in their perceived direction of effect, with some perceived to increase eating disorder risk and others perceived to decrease risk, highlighting the need to examine and deconstruct complex interventions in much more detail. Similarly, detailed examination of delivery features for differing effects is an important consideration. For example, the use of telehealth was suggested by survey respondents to differ from the broad strategy of online intervention delivery. This is due to emerging evidence finding an association between the use of telehealth that involves viewing oneself on a video screen and appearance concerns [29]. Thus, in understanding the effects of weight management interventions, it is important to deconstruct these into their smallest measurable components. The findings from this study have informed the development of a detailed coding framework to be used as part of the EDIT Collaboration (manuscript under review) and can be used to examine other safety or effectiveness outcomes (e.g., weight regain, health-related QOL, depression etc.).

The broad consultation approach of this study allowed additional complexities relating to the intersection of obesity and eating disorders to be identified. In particular, the role of weight stigma from health professionals, the types of communication and language used by health professionals and the individual variation in how such messages are experienced by different people were strong themes. The association between weight stigma and disordered eating is well established [30]; however, to our knowledge, there is no clear method that can be used to identify and measure a person’s experience of stigma as part of weight management interventions. This highlights the importance of considering individual experience as part of our understanding of treatment response and including stakeholders in setting the research agenda. Further research is needed to understand how to assess weight stigma during weight management interventions and how to consider individual variation in response.

This was the first international consultation process aiming to improve our understanding of eating disorder risk during weight management interventions. We used a rigorous development process, including expert consultation and review, allowing a comprehensive range of factors to be investigated. The inclusion of open text boxes at each stage of the survey increased the likelihood of greater coverage, and thus, more informative results. The survey had broad reach, and clinicians, researchers and people with lived experience across disciplines of overweight/obesity and eating disorders responded to the survey. Importantly, most participants reported lived experience of overweight, obesity and/or an eating disorder. Therefore, we were able to capture a diversity of views on the intersection between weight management interventions and eating disorders, i.e., some participants appear to support the notion that eating disorder risk can be considered as part of weight management, while others suggested that weight management should not occur. To our knowledge, this is the first research reporting such diversity of views and experiences. This allowed additional complexities relating to the intersection of obesity and eating disorders to be identified. There were also several limitations. Although the survey was publicized though international associations, the survey was only available in English, limiting participation to those who are fluent in the English language. Most participants were from Australia or the United States and identified as being white and women, limiting the geographic, cultural and gender diversity of participants. The sample was composed of people working across and/or with lived experience of overweight/obesity and eating disorders and are likely those with an interest in this intersecting area. We were unable to analyze differences in responses between professional groups or people with and without lived experience due to the degree of overlap in respondent background.

## 5. Conclusions

This study provides insight into the views of clinicians, researchers and people with lived experience regarding eating disorder risk during weight management interventions. Findings highlight the importance of stakeholder consultation and will inform future assessment of eating disorder risk during weight management interventions. The interaction between individual characteristics and intervention strategies identified as relevant for eating disorder risk should be examined in future research and considered in clinical practice. The EDIT Collaboration aims to address these future research questions.

## Figures and Tables

**Table 1:** Participant characteristics, n = 87.

Age, n (%)		RESEARCHERS	
18–25 years	7 (8)	Areas of research knowledge/experience/expertise,* n (%)	
26–34 years	19 (22)	Overweight/obesity	25 (29)
35–49 years	35 (40)	Binge eating disorder	20 (23)
50–64 years	18 (21)	Anorexia nervosa	17 (20)
60+ years	8 (9)	Other eating disorders (e.g., ARFID, body image, EDNOS)	17 (20)
Gender, n (%)		Bulimia nervosa	14 (16)
Female/Women	81 (93)	Atypical anorexia nervosa	13 (15)
Male/Men	4 (5)	Years focused on research area, mean (SD)	
Nonbinary	1 (1)	Overweight/obesity	10.1 (9.1)
Prefer not to say	1 (1)	Eating disorders	8.6 (8.6)
Country of residence, n (%)		Co-occurring eating disorders and overweight/obesity	6.4 (8.7)
Australia	37 (42)	Age group/s of research focus, n (%)	
USA	33 (38)	Children (<10 years)	9 (10)
UK	10 (11)	Adolescents (10–17 years)	18 (21)
Other **	7 (8)	Adults (>18 years)	19 (22)
Cultural background/ethnicity, n (%)		LIVED EXPERIENCE	
White	57 (66)	Lived experience,* n (%)	
Mixed race	4 (5)	High weight (BMI 25–39.9)	46 (53)
Other (Asian n = 3, Black n = 1, Hispanic n = 1, New Zealand Māori, n = 1)	6 (7)	High weight (BMI > 40)	11 (13)
None	19 (22)	Anorexia nervosa	17 (20)
Prefer not to say	2 (2)	Atypical anorexia nervosa	11 (13)
Professional group * n (%)		Bulimia nervosa	12 (14)
Clinician	60 (69)	Binge eating disorder	16 (18)
Researcher	32 (37)	Other Specified Feeding and Eating Disorders	14 (16)
Neither	16 (18)	Other eating disorder (e.g., ARFID, orthorexia)	7 (8)
Other	8 (9)	Carer/support for person with high weight	12 (14)
CLINICIANS		Carer/support for person with an eating disorder	14 (16)
Discipline, n (%)		Other (Both anorexia nervosa and atypical anorexia nervosa based on BMI status; mental health conditions other than eating disorder)	3 (3)
Dietitian/nutritionist	27 (31)	None of the above	16 (18)
Psychologist/clinical psychologist	13 (15)	Experience of weight management treatment, n (%)	
Pediatrician	7 (8)	Previous weight management treatment	18 (21)
Other ^	19 (22)	Currently receiving weight management treatment	4 (5)
Area of clinical experience/expertise, * n (%)		Never received weight management treatment	28 (32)
Overweight/obesity	49 (56)	Experience of eating disorder treatment, n (%)	
Binge eating disorder	45 (52)	Previous eating disorder treatment	16 (18)
Anorexia nervosa	36 (41)	Currently receiving eating disorder treatment	3 (3)
Bulimia nervosa	36 (41)	Never received eating disorder treatment	19 (22)
Atypical anorexia nervosa	32 (37)		
Other eating disorders ^^	32 (37)		
Years involved in treatment, mean (SD)			
Eating disorders	11.7 (10.1)		
Overweight/obesity	10.9 (9.1)		
Co-occurring eating disorders and overweight/obesity	9.6 (8.9)		
Hours per week involved in the treatment, mean (SD)			
Eating disorders	17.3 (15.0)		
Overweight/obesity	16.0 (15.7)		
Co-occurring eating disorders and overweight/obesity	10.2 (11.4)		
Age group/s of patient seen, n (%)			
Children (<10 years)	20 (23)		
Adolescents (10–17 years)	36 (41)		
Adults (>18 years)	44 (51)		

* Respondents could select all that apply. ** Canada n = 4, Belgium n = 1, South Africa n = 1, Mexico n = 1. ^ Endocrinologist n = 3, Social worker n = 3, Counselor n = 3, GP/primary care physician n = 2, Psychiatrist n = 2, Nurse n = 2, Obesity medicine physician n = 2, Physiotherapist n = 1, Exercise physiologist n = 1. ^^ ARFID, OSFED, subclinical eating disorder, emotional eating.

**Table 2:** Additional items suggested as being relevant to eating disorder risk in the context of weight management.

Category in Survey	Broad Theme	Specific Item
PART 1: INDIVIDUAL CHARACTERISTICS		
Family and medical history	Genetic predisposition to obesity	Genetic risk factors for obesity Genetic influence on body shape/composition—what is normal body size for the individual
	Weight status	History of weight fluctuation, including degree of weight change
	Medical history	History of bariatric surgery Investigate, diagnose and treat obstructive sleep apnea
	Experience of weight stigma from health professionals	Comments on weight status Withholding of medical care until weight loss occurs Trauma related to medical stigma Attitude of medical provider towards weight/BMI, especially for children/adolescents Stigmatizing micro-aggressions Previous attendance at support groups
Psychosocial health and eating behaviors	Executive function	Difficulty with time management (especially parents of children)
	Mental health (history or current)	Borderline personality disorder Addiction (including alcohol abuse) Self-harm, suicide attempts
	Disordered eating attitudes or behaviors	Purging Compulsive exercise Body checking Heavy use of apps/tracking food intake
	Emotional response to dieting/weight loss	Emotional response to unsuccessful weight loss Compliments for weight loss (negative, positive feedback), leading to further weight loss attempts Shame/guilt
Other	Family context	Resilience of family Family history around negative attitudes to food/weight/body shape and food being used in families for emotional manipulation Exposure to self-disparaging comments about weight, especially from parents Patient/parent relationship Cultural factors
	Minority groups (vulnerable/marginalized groups)	Power imbalance with health professionals Gender identity Athletes
	Social determinants of health (other than food insecurity)	
	Environmental context/social media use	Social media use Exposure to diet culture Body size in relation to those around them Experience of fatphobia Consider risk factors (e.g., body shape/weight disturbance) within the social context of the individual, e.g., body shape/weight disturbance is reasonable in the context of societies that deem larger bodies as less valuable
PART 2: INTERVNETION STRATEGIES		
Intervention framing	Communication approaches	Decrease emphasis on personal responsibility (decreases risk) Use encouragement (rather than measurements) Individualized framing Program language/images used, e.g., comparing to ‘success’ stories Emphasizes broader quality of life benefits rather than focusing on weight
	Education approaches	Explaining the science of weight/appetite regulation and weight stigma (decrease risk) Using life expectancy to frame weight loss as a path to health and longevity Discussing weight in terms of healthy growth
Dietary strategies	Role of family dynamics in food choice/selection	Consider food insecurity as part of intervention Food choices differ from rest of household Family education on eating disorder risk and flexible eating
	Framing/labeling of foods	Framing/delivery/communication of all dietary strategies is important Consider how foods feel in the body, energy levels, satiation Use of good/bad language, e.g., dichotomizing food as “healthy” vs. “unhealthy” (increase risk)
	Dietary prescription	Rigid dietary prescriptions increase risk Weighing and measuring foods (increases risk)
Eating behavior strategies	Role of culture	Consider cultural context of food and role of food in culture (decrease risk).
	Address energy restriction	Energy restriction leading to hunger and binge eating (cycle) (increases risk). Education on a healthy menstrual cycle and link with under-eating and over-exercising.
	Context of eating behavior	Use of intuitive eating in the context of weight management increases eating disorder risk. Emotional eating is a normal part of intuitive and mindful eating and should not be demonized or categorized as disordered eating.
	Comments re eating behaviors	Examining disordered eating behaviors may not be appropriate in children (safety concern?) Screening for these eating behaviors may help to identify eating disorders and facilitate referral and support. Screening is unlikely to exacerbate disordered eating.
Movement and sleep strategies	Sleep quality	Improve circadian alignment and total sleep time
	Attitudes, beliefs and training/qualifications of trainers contributes to level of ED risk	Appearance- and weight-loss-focused personal trainer (increase risk) Focus on healthy habits, QOL, goals—unlikely to increase risk
	Motivations for activity	Mental and physical health motivations for exercise vs. shape/weight/appearance motivators Promoting PA/movement without a weight loss focus (decreases risk). Having exercise focused on weight loss rather than being fun.
Psychosocial health	Education/approach to aspects of psychosocial health	Education on internalized and externally received weight biases. Health at Every Size principles Education that undereating is related to anxiety, depression and difficulty concentrating. Education on difficulty with maintaining long term weight loss—sense of failure can lead to shame. Strategies that utilize normalizing and addressing shame for carer and young person
PART 3: DELIVERY FEATURES		
Duration of intervention	Need for long term support/continuity of care	Weight regain can lead to self-blame, feelings of failure Step up/step down pathways Multidisciplinary care
Mode of delivery	Mode of delivery	Telehealth/video conferencing associated with appearance concerns and body dissatisfaction
Setting	Environment	Environmental contributions, e.g., supportive chairs Safe space without intersectional biases. Understanding of group rules and behavior
Personnel	Training needs	Professionals (providing weight management) need to have a thorough understanding of ED and body image issues

* Comments have been categorized based on those used in the original survey even if reported in a different section. Where direction of risk was noted in the comment, this has been included. For most items, proposed direction of risk was not suggested.

## Data Availability

The data presented in this article are not publicly available.

## Supplementary Materials

The following supporting information can be downloaded at https://www.mdpi.com/article/10.3390/nu15051085/s1, Table S1: Individual participant characteristics; Table S2: Category 1: Framing of the Intervention; Table S3: Category 2: Dietary strategies; Table S4: Category 3: Eating behaviors/disordered eating; Table S5: Category 4: Movement and sleep related strategies; Table S6: Category 5: Psychosocial health related strategies; Table S7: Delivery Features (n = 67).
