# Supplementary material for: Identifying Factors Which Influence Eating Disorder Risk during Behavioral Weight Management: A Consensus Study
Source: Nutrients. 2023 Feb 22;15(5):1085. doi: 10.3390/nu15051085 (PMC10005214; doi:10.3390/nu15051085)
Supplement: Supplementary file 1 [file nutrients-15-01085-s001.zip › nutrients-2212347-supplementary.pdf]

Table S1: Individual participant characteristics

Individual characteristics (n=87)						
	N (%)					
	Very unlikely to be relevant	Unlikely to be relevant	Somewhat likely to be relevant	Likely to be relevant	Very likely to be relevant	Not sure
**Participant demographics** e.g. age, sex, ethnicity, socio-economic status, family structure and environment	3 (3)	3 (3)	16 (18)	23 (26)	39 (45)	3 (3)
**Weight status or classification** e.g. BMI, BMI z-score, BMI percentile, overweight, obesity	7 (8)	11 (13)	11 (13)	18 (21)	37 (43)	3 (3)
**General medical history (excluding mental health) (n=87)**						
Age at menarche	8 (9)	5 (6)	26 (30)	34 (39)	7 (8)	7 (8)
Age at menopause	11 (13)	19 (22)	17 (20)	19 (22)	5 (6)	16 (18)
Medical history e.g., type 2 diabetes, diet-related chronic disease	1 (1)	11 (13)	24 (28)	30 (35)	19 (22)	2 (2)
Family history e.g., type 2 diabetes, diet-related chronic disease	1 (1)	13 (15)	29 (33)	24 (28)	15 (17)	5 (6)
Medication history -general	4 (5)	15 (17)	24 (28)	28 (32)	4 (5)	12 (14)
Medication history - those known to effect body weight	2 (2)	3 (3)	15 (17)	34 (39)	28 (32)	5 (6)
Family history of bariatric surgery	1 (1)	6 (7)	15 (17)	33 (38)	27 (31)	5 (6)
**Weight-related medical history (n=84)**						
Family history of obesity	2 (2)	1 (1)	9 (11)	31 (37)	39 (46)	2 (2)
Recent weight loss prior to intervention	0 (0)	6 (7)	12 (14)	30 (36)	33 (39)	3 (4)
Age of onset of obesity/ weight history	2 (2)	4 (5)	9 (11)	29 (35)	35 (42)	5 (6)
Life events which may impact upon body weight e.g. pregnancy, smoking cessation	1 (1)	6 (7)	12 (14)	32 (38)	27 (32)	6 (7)
**Eating disorder-related medical history (n=84)**						
History of an eating disorder (current, previous)	0 (0)	1 (1)	2 (2)	7 (8)	74 (88)	0 (0)
History of eating disorder treatment	1 (1)	1 (1)	3 (4)	14 (17)	65 (77)	0 (0)
Family history of eating disorder	1 (1)	1 (1)	4 (5)	15 (18)	63 (75)	0 (0)
**Mental health-related medical history (n=83)**						
Diagnosis of anxiety or depression	0 (0)	3 (4)	6 (7)	29 (35)	44 (53)	1 (1)
Trauma (including adverse childhood experience, trauma during adulthood)	0 (0)	3 (4)	1 (1)	22 (27)	57 (69)	0 (0)
Diagnosis of oppositional defiant disorder (ODD)	5 (6)	14 (17)	11 (13)	12 (15)	14 (17)	27 (33)
Diagnosis of attention deficit hyperactivity disorder (ADHD)	3 (4)	7 (8)	19 (23)	17 (21)	19 (23)	18 (22)
Diagnosis of obsessive compulsive disorder (OCD)	2 (2)	3 (4)	7 (8)	27 (33)	39 (47)	5 (6)
Diagnosis of autism spectrum disorder (ASD)	2 (2)	8 (10)	18 (22)	18 (22)	20 (24)	17 (21)
Personality traits e.g. perfectionism, obsessionality, impulsivity, unrelenting standards	1 (1)	2 (2)	1 (1)	21 (25)	56 (68)	2 (2)

Psychosocial health at treatment outset (n=83)						
Self-esteem	1 (1)	2 (2)	8 (10)	18 (22)	53 (64)	1 (1)
Body dissatisfaction	0 (0)	1 (1)	4 (5)	8 (10)	69 (83)	1 (1)
Weight-bias internalisation	0 (0)	1 (1)	2 (2)	11 (13)	68 (82)	1 (1)
Weight-based teasing/bullying/ stigma/discrimination	0 (0)	1 (1)	2 (2)	9 (11)	70 (84)	1 (1)
Weight-talk (peer/family)	0 (0)	1 (1)	6 (7)	15 (18)	59 (71)	2 (2)
Quality of life/ weight-related quality of life	0 (0)	1 (1)	8 (10)	23 (28)	49 (59)	2 (2)
Stress	0 (0)	2 (2)	15 (18)	23 (28)	42 (51)	1 (1)
Social support (family support/ peer support)	0 (0)	2 (2)	15 (18)	26 (31)	38 (46)	2 (2)
**Eating behaviours at treatment (n=80)**						
Disinhibition related to eating	5 (6)	2 (3)	5 (6)	25 (31)	37 (46)	6 (8)
Impulsivity related to eating	3 (4)	2 (3)	6 (8)	21 (26)	46 (58)	2 (2)
Emotional eating	4 (5)	4 (5)	6 (8)	18 (23)	47 (59)	1 (1)
Binge eating	2 (3)	2 (3)	1 (1)	17 (21)	57 (71)	1 (1)
Loss of control eating	2 (3)	2 (3)	2 (3)	15 (19)	58 (73)	1 (1)
Self-efficacy towards eating	3 (4)	3 (4)	8 (10)	27 (34)	35 (44)	4 (5)
Night eating	3 (4)	4 (5)	11 (14)	24 (30)	36 (45)	2 (3)
Grazing	5 (6)	17 (21)	22 (28)	15 (19)	18 (23)	3 (4)
External eating/food responsiveness (i.e., eating in response to external cues)	5 (6)	9 (11)	10 (13)	18 (23)	34 (43)	4 (5)
Secret eating	2 (3)	2 (3)	2 (3)	16 (20)	57 (71)	1 (1)
Family history or current food insecurity or deprivation	1 (1)	2 (3)	10 (13)	18 (23)	46 (58)	3 (4)
Narrow range/limited food choices or acceptability (including food rules, limited access to foods, fussy eating)	0 (0)	1 (1)	11 (14)	22 (28)	43 (54)	3 (4)
**History of dieting (n=80)**						
Level of dietary restraint/restriction	0 (0)	3 (4)	5 (6)	16 (20)	55 (69)	1 (1)
Previous dieting under professional supervision	3 (4)	9 (11)	16 (20)	19 (24)	32 (40)	1 (1)
Previous self-directed dieting	0 (0)	2 (3)	13 (16)	18 (23)	46 (58)	1 (1)
Type of diet followed during dieting	1 (1)	7 (9)	9 (11)	21 (26)	41 (51)	1 (1)
Duration of dieting	2 (3)	4 (5)	11 (14)	19 (24)	43 (54)	1 (1)
Number of previous dieting attempts	2 (3)	4 (5)	8 (10)	17 (21)	47 (59)	2 (3)
Age of onset of dieting	1 (1)	2 (3)	6 (8)	20 (25)	49 (61)	2 (3)

Part 2: Weight Management Strategy Interventions

Table S2.1. Category 1: Framing of the Intervention

Intent/overarching goal of the weight management intervention (n=71)						
	N (%)					
	Very likely to decrease risk of eating disorder	Somewhat likely to decrease risk	No impact on eating disorder risk	Somewhat likely to increase risk	Very likely to increase risk of eating disorder	Not sure

Aims for weight maintenance but not weight loss	3 (4)	19 (27)	11 (15.5)	18 (25)	18 (25)	2 (3)
Aims for weight loss	3 (4)	1 (1)	7 (10)	21 (30)	38 (54)	1 (1)
Does not focus on weight in any way, focus on health improvement e.g. a weight-neutral intervention	26 (37)	25 (35)	10 (14)	6 (9)	2 (3)	2 (3)
**Framing of the intervention (communication strategies) (n=71)**						
Education provided on obesity as a disease	3 (4)	20 (28)	6 (9)	17 (24)	23 (32)	2 (3)
Education that weight loss is required to improve health outcomes	1 (1)	11 (16)	5 (7)	21 (30)	31 (44)	2 (9)
Education that health outcomes are not dependent on weight	19 (27)	33 (47)	10 (14)	2 (3)	2 (3.0)	5 (7)
Use of weight-focused language during the intervention (e.g., weight loss, weight management, weight control)	0 (0)	6 (9)	9 (13)	18 (25)	35 (49)	3 (4)
Feedback on change in metabolic health outcomes (e.g., insulin sensitivity, cholesterol levels)	8 (11)	22 (31)	19 (27)	12 (17)	4 (6)	6 (9)
**Outcome related strategies (n=69)**						
Measures weight/ adiposity as an outcome e.g. weight, waist circumference, body composition	1 (1)	4 (6)	6 (9)	25 (36)	32 (46)	1 (1)
Measures a range of health outcomes e.g. blood pressure, blood test, fitness	7 (10)	23 (33)	14 (20)	15 (22)	5 (7)	5 (7)
Measures mental health outcomes e.g. depression	12 (17)	31 (45)	10 (15)	7 (10)	7 (10)	2 (3)
Encourages weight focused goals	1 (1)	3 (4)	4 (6)	18 (26)	42 (61)	1 (1)
Discourages weight-focused goals (instead focused on health-related goals)	17 (25)	32 (46)	10 (15)	8 (12)	1 (1)	1 (1)
Feedback on weight change during the intervention	2 (3)	4 (6)	10 (15)	18 (26)	30 (44)	5 (7)
Feedback on other measures of weight adiposity (e.g. body composition, waist circumference)	3 (4)	10 (15)	9 (13)	13 (19)	30 (44)	4 (6)
Promotes weight loss rewards or incentives	2 (3)	2 (3)	6 (9)	15 (22)	43 (62)	1 (1)
**Measurement of weight (n=69)**						
Individual weighing at visits	1 (1)	6 (9)	8 (12)	20 (29)	34 (49)	0 (0)
Blind weighing at visits	5 (7)	13 (19)	16 (23)	20 (29)	11 (16)	4 (6)
Group weighing	1 (1)	1 (1)	1 (1)	17 (25)	48 (70)	1 (1)
Encourages self-monitoring of weight (e.g. self-weighing at home)	0 (0)	6 (9)	9 (13)	16 (23)	37 (54)	1 (1)

Discourages home weighing or frequent weighing	6 (9)	36 (52)	17 (25)	2 (3)	5 (7)	3 (4)
Communication about the ability to decline weight or opt out of weighing during visits	8 (12)	33 (48)	16 (23)	4 (6)	4 (6)	4 (6)

Table S2.2. Category 2: Dietary strategies

**Nutrition education (n=64)**						
	**N** (%)					
	**Very likely to decrease risk of eating disorder**	**Somewhat likely to decrease risk**	**No impact on eating disorder risk**	**Somewhat likely to increase risk**	**Very likely to increase risk of eating disorder**	**Not sure**
Education on portion size (e.g. portion plate model)	3 (5)	10 (16)	15 (23)	21 (33)	13 (20)	2 (3)
Education on label reading	3 (5)	8 (13)	16 (25)	19 (30)	16 (25)	2 (3)
Education on metabolism	8 (13)	20 (31)	17 (27)	10 (16)	7 (11)	2 (3)
Education on healthy eating guide (e.g. promotes balanced meals and food groups)	8 (13)	21 (33)	18 (28)	10 (16)	6 (9)	1 (2)
Education on energy/ macronutrient (e.g. fat, sugar) content of foods	6 (10)	5 (8)	14 (22)	25 (39)	13 (20)	1 (2)
Categorisation of foods (e.g. traffic light system; increase certain foods such as fruit and vegetables; reduce/limit certain foods such as alcohol, high energy, high fat, high sugar; defines foods as good vs bad (e.g treat/ sometimes foods)	5 (8)	4 (6)	4 (6)	21 (33)	30 (47)	0 (0)
Provides cultural adaptations relating to diet	8 (13)	19 (30)	26 (41)	5 (8)	4 (6)	2 (3)
**Dietary monitoring (n=64)**						
Dietary monitoring – food based (e.g. food diary, points system)	3 (5)	3 (5)	7 (11)	15 (23)	34 (53)	2 (3)
Dietary monitoring – energy based (e.g. calorie counting)	2 (3)	2 (3)	1 (2)	16 (25)	42 (66)	1 (2)
Review/feedback on self-monitoring (e.g., feedback on food diary)	3 (5)	7 (11)	8 (13)	13 (20)	25 (39)	8 (13)
**Dietary prescription (n=64)**						
Hypocaloric diet (reduced calorie diet)	1 (2)	3 (5)	5 (8)	13 (20)	41 (64)	1 (2)
Traffic light diet (categorising foods as red, yellow, green)	2 (3)	6 (9)	4 (6)	22 (34)	29 (45)	1 (2)
Intermittent energy restriction/ intermittent fasting (chrononutrition)	1 (2)	2 (3)	6 (9)	7 (11)	45 (70)	3 (5)
Macronutrient prescription (e.g. low-carbohydrate, high-protein)	3 (5)	1 (2)	8 (13)	16 (25)	33 (52)	3 (5)

Ketogenic diet	1 (2)	2 (3)	5 (8)	12 (19)	36 (56)	8 (13)
Moderate energy restriction (moderate calorie restriction)	3 (5)	5 (8)	7 (11)	21 (33)	26 (41)	2 (3)
Very low energy diet (VLED/ VLCD - restrictive calorie restriction e.g. 800-1000 kcal/ day)	0 (0)	3 (8)	3 (5)	9 (14)	46 (72)	3 (5)
**Delivery of the dietary intervention (n=64)**						
Prescriptive/ specific meal plan (external control)	3 (5)	4 (6)	5 (8)	18 (28)	30 (47)	4 (6)
Flexible meal plan (provides choice, ownership over dietary intake)	12 (19)	27 (42)	8 (13)	8 (13)	7 (11)	2 (3)
Use of meal replacement products – partial or full	2 (3)	5 (8)	9 (14)	21 (33)	23 (36)	4 (6)
Promotes ‘free’ foods, ad-lib intake of certain foods	2 (3)	13 (20)	7 (11)	16 (25)	19 (30)	7 (11)
Individual approach to dietary change	15 (23)	28 (44)	7 (11)	6 (9)	7 (11)	1 (2)
Family-oriented approach to dietary change	14 (22)	27 (42)	4 (6)	8 (13)	8 (13)	3 (5)
**Dietary behaviour change strategies (n=63)**						
Problem solving barriers to dietary change	10 (16)	26 (41)	7 (11)	7 (11)	6 (10)	7 (11)
Feedback on dietary behaviours (e.g. diet history at visits)	6 (10)	13 (21)	12 (19)	14 (22)	13 (21)	5 (8)
Encourages dietary focused goals with/ without review	3 (5)	14 (22)	16 (25)	14 (22)	9 (14)	7 (11)
Shopping support (planning, product choice, family/ partner involvement in food purchases)	11 (18)	20 (32)	14 (22)	6 (10)	7 (11)	5 (8)
Addresses home/food environment (e.g. identifying triggers, permissive vs restrictive environment; stimulus control)	12 (19)	30 (48)	5 (8)	6 (10)	8 (13)	2 (3)
Addresses food/meal preparation skills (e.g. cooking demonstrations, recipes)	10 (16)	29 (46)	12 (19)	3 (5)	7 (11)	2 (3)

Table S2.3. Category 3: Eating behaviors/disordered eating

**Addresses disordered eating (n=63)**						
	**N** (%)					
	**Very likely to decrease risk of eating disorder**	**Somewhat likely to decrease risk**	**No impact on eating disorder risk**	**Somewhat likely to increase risk**	**Very likely to increase risk of eating disorder**	**Not sure**
Identifies disordered eating behaviours (e.g. binge eating, emotional eating, secret eating, guilt related to eating, loss of control over eating)	19 (30)	26 (41)	8 (13)	2 (3)	7 (11)	1 (2)

Explores individual underlying causes/ drivers of disordered eating (e.g. teasing/ bullying, trauma, body image disturbance/pre-occupation with weight and shape, emotional regulation)	27 (43)	25 (40)	5 (8)	1 (2)	4 (6)	1 (2)
Addresses disordered eating behaviours (e.g. identifying triggers, strategies to prevent emotional eating)	31 (49)	19 (30)	2 (3)	2 (3)	7 (11)	2 (3)
**Promotes healthful/ helpful eating behaviours (n=63)**						
Promotes mealtime routines (e.g. regular meals, avoid meal skipping)	23 (37)	31 (49)	2 (3)	2 (3)	5 (8)	0 (0)
Promotes meal time support (e.g. support while eating, family meals, social eating)	18 (29)	32 (51)	6 (10)	1 (2)	5 (8)	1 (2)
Encourages mindful eating principles or practice (e.g. avoiding distractions while eating)	21 (33)	26 (41)	9 (14)	2 (3)	4 (6)	1 (2)
Encourages intuitive eating principles or practice (e.g. Promotes anti-diet, hunger and fullness, food enjoyment, body respect)	27 (43)	26 (41)	3 (5)	2 (3)	4 (6)	1 (2)
Increasing awareness of hunger/ fullness/ satiety	25 (40)	29 (46)	4 (6)	2 (3)	3 (5)	0 (0)

Table S2.4. Category 4: Movement and sleep related strategies

**Physical activity education (n=55)**						
	**N** (%)					
	**Very likely to decrease risk of eating disorder**	**Somewhat likely to decrease risk**	**No impact on eating disorder risk**	**Somewhat likely to increase risk**	**Very likely to increase risk of eating disorder**	**Not sure**
Education to increase physical activity (e.g. staged introduction of activity, suggested activities)	5 (9)	18 (33)	13 (24)	12 (22)	5 (9)	2 (4)
Promotes joyful movement and activity	16 (29)	29 (53)	2 (4)	4 (7)	3 (6)	1 (2)
Encourages strict/ formal activity plan (e.g. gym program)	1 (2)	4 (7)	5 (9)	26 (47)	18 (33)	1 (2)
Education on Non Exercise Activity Thermogenesis (NEAT) (energy expended during tasks of daily living)	3 (6)	19 (35)	10 (18)	8 (15)	7 (13)	8 (15)
Provides cultural adaptations relating to physical activity	11 (20)	23 (42)	13 (24)	2 (4)	4 (7)	2 (4)

**Physical activity prescription (n=55)**						
Provides a prescriptive exercise plan	3 (6)	4 (7)	4 (7)	22 (40)	18 (33)	4 (7)
Provides flexible exercise plan (e.g. suggested activities, encouraging choice)	7 (13)	31 (56)	7 (13)	6 (11)	3 (6)	1 (2)
Provides supervised group exercise classes/ program	5 (9)	20 (36)	10 (18)	8 (15)	6 (11)	6 (11)
Provides individual personal training	4 (7)	12 (22)	10 (18)	12 (22)	5 (9)	12 (22)
**Physical activity monitoring (n=55)**						
Self-monitoring of activity (e.g. diary, pedometer)	3 (6)	4 (7)	6 (11)	22 (40)	15 (27)	5 (9)
External feedback on self-monitoring (e.g., feedback on exercise diary, step count)	3 (6)	3 (6)	9 (16)	16 (29)	17 (31)	7 (13)
**Behaviour change strategies related to physical activity (n=55)**						
Encourage activity focused goals (including time, duration, mode) with/ without review	4 (7)	8 (15)	9 (16)	20 (36)	9 (16)	5 (9)
Increasing skills to undertake physical activity (e.g. demonstration of activity such as pictures/ videos/ live demos, help with scheduling/ planning for activity)	6 (11)	13 (24)	16 (29)	6 (11)	6 (11)	8 (15)
Feedback on physical activity behaviours and/ or change in fitness	7 (13)	12 (22)	13 (24)	10 (18)	9 (16)	4 (7)
Problem solving barriers to physical activity	8 (15)	22 (40)	13 (24)	3 (6)	5 (9)	4 (7)
**Addressing sedentary time (n=54)**						
Education to reduce/ limit sedentary time (e.g. screen time)	2 (4)	10 (19)	22 (41)	10 (19)	8 (15)	2 (4)
Sedentary time focused goals (time, duration, mode) with/ without review	2 (4)	11 (20)	17 (32)	13 (24)	7 (13)	4 (7)
Encourages self-monitoring of sedentary time (e.g. screen time monitoring, setting app limits, reminders to stand)	3 (6)	6 (11)	19 (35)	13 (24)	10 (19)	3 (6)
Problem solving barriers to reducing sedentary time	5 (9)	14 (26)	20 (37)	7 (13)	5 (9)	3 (6)
**Addressing sleep health (n=54)**						
Promotes regular sleep routine	12 (22)	25 (46)	12 (22)	0 (0)	3 (6)	2 (4)
Promotes positive sleep environment (e.g. limit screen time before bed)	9 (17)	27 (50)	13 (24)	0 (0)	3 (6)	2 (4)

Table S2.5. Category 5: Psychosocial health related strategies

Psychological framework or theory (n=55)						
	N (%)					
	Very likely to decrease risk of eating disorder	Somewhat likely to decrease risk	No impact on eating disorder risk	Somewhat likely to increase risk	Very likely to increase risk of eating disorder	Not sure
Intervention is informed by a psychological framework or theory	9 (16)	22 (40)	6 (11)	0 (0)	8 (15)	10 (18)
Intervention is based on Cognitive behaviour therapy (CBT)	6 (11)	29 (53)	4 (7)	3 (6)	8 (15)	5 (9)
Intervention is based on Enhanced Cognitive Behaviour Therapy (CBT-E)	11 (20)	25 (46)	2 (4)	2 (4)	8 (15)	7 (13)
Intervention is based on Acceptance and Commitment therapy (ACT)	10 (18)	24 (44)	5 (9)	1 (2)	7 (13)	8 (15)
Intervention is based on Dialectical Behaviour Therapy (DBT)	7 (13)	23 (42)	5 (9)	1 (2)	7 (13)	12 (22)
Intervention is based on Family-Based Treatment	7 (13)	30 (55)	2 (4)	1 (2)	8 (15)	7 (13)
Intervention is based on Interpersonal Therapy (IPT) for binge eating	6 (11)	23 (42)	2 (4)	1 (2)	8 (15)	15 (27)
Intervention is based on Trauma-informed care	12 (22)	29 (53)	4 (7)	1 (2)	5 (10)	4 (7)
Intervention is based on Compassion focussed therapy	10 (18)	26 (47)	5 (9)	1 (2)	7 (13)	6 (11)
Intervention uses Motivational interviewing	6 (11)	26 (47)	9 (16)	1 (2)	8 (15)	5 (9)
**Addresses mental health conditions e.g. depression, anxiety, PTSD (n=55)**						
Identifies mental health condition	10 (18)	25 (46)	11 (20)	2 (4)	4 (7)	3 (6)
Provides referral for psychological support	22 (40)	21 (38)	5 (9)	1 (2)	5 (9)	1 (2)
Addresses mental health condition within the intervention	19 (35)	23 (42)	6 (11)	1 (2)	5 (9)	1 (2)
Addresses self-esteem	19 (35)	24 (44)	7 (13)	1 (2)	4 (7)	0 (0)
**Addresses body image (n=55)**						
Addresses body image concerns	18 (33)	25 (46)	4 (7)	2 (4)	4 (7)	2 (4)
Education on the role of social media (e.g. media literacy training)	15 (27)	28 (51)	3 (6)	1 (2)	5 (9)	3 (6)
Promotes body compassion/ acceptance/ positivity	21 (38)	21 (38)	4 (7)	2 (4)	5 (9)	2 (4)
**Addresses weight stigma (n=55)**						

Education and/ or strategies to increase resilience to weight stigma, bullying, teasing	16 (29)	21 (38)	10 (18)	1 (2)	4 (7)	3 (6)
Addresses weight-focused communication skills (e.g. how to communicate with peers/ family about weight, how to address weight-related comments from peers/ family)	18 (33)	22 (40)	8 (15)	0 (0)	4 (7)	3 (6)
**Psychosocial health related monitoring (n=55)**						
Self-monitoring of thoughts, feelings, mood (e.g. mood diary)	7 (13)	29 (53)	12 (22)	2 (4)	3 (6)	2 (4)
Review/feedback on self-monitoring (e.g. mood diary)	7 (13)	30 (55)	11 (20)	0 (0)	4 (7)	3 (6)
Encourages self-assessment of overall wellbeing (e.g. reflective practice)	12 (22)	30 (55)	5 (9)	1 (2)	5 (9)	2 (4)
**Behaviour change strategies related to psychosocial issues (n=55)**						
Encourages psychosocial health related goals with/ without review	6 (11)	31 (57)	4 (7)	2 (4)	5 (9)	7 (13)
Increases skills to manage psychosocial health (e.g. stress management)	15 (27)	29 (53)	4 (7)	1 (2)	4 (7)	2 (4)
Inclusion of peer/ social support strategies	13 (24)	29 (53)	5 (9)	0 (0)	5 (9)	3 (6)

Table S3: Delivery Features (n=67)

Delivery Feature	N (%)		
	Not important	Important	Unsure
Who delivered the intervention, including profession, training and qualifications	9 (13)	54 (81)	4 (6)
Setting of the intervention e.g. primary care, hospital, community	22 (33)	35 (52)	10 (15)
Mode of delivery e.g. Face-to-face, online, SMS, telehealth	19 (28)	33 (49)	15 (22)
Target population/ recipient of the intervention e.g. group or individual intervention, whether intervention is delivered to individual alone or with support person such as a partner or parent	10 (15)	44 (66)	13 (19)
Support provided during the intervention e.g. Frequency and duration of contact with intervention personnel	5 (8)	55 (82)	7 (10)
